# From Mechanism to Therapy: Isoliquiritigenin as a Novel Anti-Inflammatory Agent for Inflammatory Disease Management

**DOI:** 10.2174/0118715303395815250904075148

**Published:** 2026-03-19

**Authors:** Fangfang Qi, Hongjin Chen, Shuhao Li, Ying Zhang, Xiong Chen, Aifang Wang

**Affiliations:** 1 Department of Pharmacology, Quzhou Maternal and Child Health Care Hospital, Quzhou, Zhejiang, China;; 2 Guizhou Province Key Laboratory of Regenerative Medicine, Tissue Engineering and Stem Cell Experiment Center, Guizhou Medical University, Guiyang, 561113, Guizhou, China;; 3 Department of Pharmacology, School of Basic Medical Sciences, Guizhou Medical University, Guiyang, 561113, Guizhou, China;; 4 Department of Endocrinology, The People's Hospital of Yuhuan, The Yuhuan Branch of The First Affiliated Hospital of Wenzhou Medical University, Yuhuan, 317600, Zhejiang, China

**Keywords:** Isoliquiritigenin, acute inflammation, chronic inflammation, cell signaling, neurological disorders, bioactive flavonoid

## Abstract

**Introduction:**

Accumulating evidence has multilaterally proved the indispensable contribution of inflammation in mediating various diseases over the last decade, including sepsis, obesity, diabetes, and neurological disorders. This established correlation between inflammation and disease progression has positioned anti-inflammatory intervention as a promising therapeutic strategy for disease prevention and treatment. Naturally occurring flavonoids have emerged as a subject of extensive investigation due to their well-documented anti-inflammatory properties and molecular mechanisms. The current review provides a comprehensive analysis of isoliquiritigenin (ISL), a bioactive flavonoid compound isolated from *Glycyrrhiza glabra* (licorice), with particular emphasis on its pharmacological activities and molecular mechanisms in modulating inflammation-associated disorders.

**Methods:**

A systematic literature review was executed across the PubMed and Google Scholar electronic databases spanning the period from January 2000 to December 2024, employing the following keyword combination: “isoliquiritigenin” (MeSH) AND “inflammation” (MeSH).

**Results:**

ISL was found to exhibit significant therapeutic potential in mitigating both acute and chronic inflammatory responses. Particular attention was devoted to elucidating ISL's multi-target regulatory mechanisms in acute organ injury models, including neurological, pulmonary, hepatic, and renal systems. Furthermore, the compound's therapeutic effects were found to extend to chronic inflammatory pathologies associated with metabolic and neurodegenerative disorders, notably diabetes mellitus, obesity-related complications, and Alzheimer's disease-associated tissue damage, particularly manifesting in ocular, pulmonary, and cardiovascular systems. Systematic characterization of ISL's molecular targets and associated signalling cascades, like MAPK, JAK/STAT3, Nrf2, and SIRT1 pathways, substantially enhanced our mechanistic understanding of its anti-inflammatory properties.

**Discussion:**

ISL demonstrated extensive protection in many inflammatory models. Its multi-target action implied broad therapeutic applicability. However, despite its excellent anti-inflammatory efficacy and safety profile, further study is required to investigate its effectiveness for clinical translation.

**Conclusion:**

This comprehensive analysis has provided a pharmacological foundation for developing ISL-based therapeutic interventions against inflammation-driven human pathologies.

## 1 INTRODUCTION

Inflammation is a classic vascular-based biological response, activated by a complex series of signaling pathways designed to protect the body from harmful agents and damaging signals or molecules. This self-defence mechanism, regulated by myeloid immune cells, is characterised by the symptoms of redness, heat, pain, swelling, and functional breakdown [1-4]. Inflammation is broadly classified into acute and chronic forms. Acute inflammation protects the body by stabilising the injured site and enabling the immune system to repair damaged tissue. In contrast, chronic inflammation exerts a detrimental effect. It continuously activates immune cells through the bloodstream, amplifying the inflammatory response. This long-term activation often triggers systemic defences, which can inadvertently damage healthy tissue and contribute to the development of serious chronic diseases. Substantial evidence derived from epidemiological observations, genetic association studies, and pharmacological interventions has consistently demonstrated a robust link between inflammation and the pathogenesis of various chronic disorders [5-7].

The initiation of inflammatory responses is triggered through the recognition of pathogen-associated molecular patterns (PAMPs) and danger-associated molecular patterns (DAMPs) by pattern-recognition receptors (PRRs), which are strategically localized at cellular membranes or within intracellular compartments [1, 4]. Among the extensively characterized PRRs, the toll-like receptor (TLR) family represents the most comprehensively studied group, with distinct membrane localization patterns encompassing both cell surface and endolysosomal compartments. The molecular interaction between TLRs and their cognate ligands initiates a well-orchestrated inflammatory response, characterized by the activation of intricate intracellular signaling cascades. These signaling events subsequently converge on the activation of canonical inflammatory pathways, notably the nuclear factor-kappa B (NF-κB), activator protein 1 (AP-1), and interferon regulatory factor (IRF) pathways. The downstream consequences of these pathway activations encompass the transcriptional upregulation of pro-inflammatory mediators, including nitric oxide (NO), prostaglandin E2 (PGE2), tumor necrosis factor-alpha (TNF-α), interleukin-1 beta (IL-1β), and interleukin-6 (IL-6), coupled with the induced expression of inflammatory enzymes, such as inducible nitric oxide synthase (iNOS) and cyclooxygenase-2 (COX-2) [8-10].

The activation of intracellular protein complexes, known as inflammatory vesicles, can trigger an inflammatory response. This response is characterised as the formation of reactive oxygen species (ROS) and the induction of autophagy [11, 12]. Two classes of inflammatory vesicles have been identified. The first category, known as typical inflammasomes, comprises NLRs and AIM2 inflammasomes, whereas another category, referred to as atypical inflammasomes, includes caspases-4, -5, and -11. Different ligands activate inflammatory vesicles by assembling inflammatory vesicle components to induce an inflammatory response [4, 13-15].

Thus, anti-inflammatory treatment has become crucial during the process of curing chronic diseases. Nonetheless, most available treatments focus on modulating specific single targets, which can lead to high costs and significant side effects, especially with prolonged use [16-19]. Considering the affordability and safety of long-term use, drugs with ingredients derived from nature have more potential for development [20, 21]. Notably, recent studies have revealed that nearly 70% of drugs that have been developed in the past 40 years have originated from natural products [22].

*Glycyrrhiza glabra*, commonly known as licorice, represents a commercially valuable botanical species with significant global economic importance, finding extensive applications across diverse industries, including pharmaceutical development, cosmetic formulations, food technology, and tobacco processing [23]. To date, the study of phytochemistry and pharmaceutical analysis relative to liquorice have been extensively carried out [24-28], and ISL, a flavonoid compound with a chalcone structure extracted from liquorice roots, has been discovered to demonstrate a variety of biological attributes, including anti-inflammatory, antioxidant, anti-angiogenic, anti-bacterial, and anti-tumour growth activities [29-33]. This review analyzes the role of ISL in different models of inflammation-associated diseases and its mechanisms to provide a resource for future clinical and basic research in the domain of anti-inflammatory therapy.

## 2 METHODOLOGY

### 2.1 Search Strategy

A structured knowledge mapping protocol was methodically applied to core biomedical repositories (PubMed/Google Scholar), spanning from the onset of the millennium to the recent end of the current decade (2000-2024), deploying advanced semantic search architectures with controlled vocabulary annotations: (isoliquiritigenin) ∩ (inflammation).

### 2.2 Study Selection

Systematic reviews and disease categories with fewer than five supporting studies were excluded to ensure methodological rigor and evidence reliability.

### 2.3 Data Extraction and Synthesis

Following the application of exclusion criteria, the relevant papers were obtained, and data extraction was performed using a standardized template that documented study designs, interventions, outcomes, and key findings. Three researchers independently conducted data extraction, with discrepancies resolved through consensus or third-party arbitration. A draft manuscript was subsequently synthesized, which underwent iterative review and finalization by three additional researchers.

## 3 ISL AND ACUTE INJURY

### 3.1 Acute Neurological Injury

Cerebral stroke usually occurs when blood flow is blocked, and it is generally divided into different categories, such as ischaemic stroke and haemorrhagic stroke [34]. It is the second lethal cause of death among adults worldwide [35]. Hitherto, lots of research studies have revealed the pivotal role of accelerating brain damage in the occurrence of stroke [36]. Therefore, it is imperative to probe into the inflammation-related pathological mechanisms in order to offer novel therapy for curing stroke. Mechanistic studies have revealed that ISL promotes microglial polarization towards the M2 phenotype through inhibition of the p38/mitogen-activated protein kinase (p38/MAPK) signaling pathway, suggesting its therapeutic potential in ischemic stroke management [37].

Spontaneous cerebral hemorrhage, representing 15-20% of all stroke cases, constitutes a severe cerebrovascular disorder characterized by high morbidity and mortality rates, posing significant threats to patient survival and neurological outcomes [38, 39]. When a blood vessel in the brain ruptures, the released blood components and cellular debris can cause severe secondary brain damage. The pathological manifestations encompass a spectrum of neurological impairments, including neurobehavioral deficits, cerebral edema, neuronal cell death, and disruption of blood-brain barrier integrity. Despite significant progress in understanding the mechanisms of brain injury after cerebral haemorrhage, there is still an absence of an effective method to prevent it. Furthermore, accelerating evidence implies that inflammatory responses and oxidative stress play a critical role in early brain dysfunction following cerebral haemorrhage [38, 40-43]. Zeng *et al.* induced experimental cerebral haemorrhage in rats and found that intraperitoneal injection of ISL after cerebral haemorrhage attenuated early brain injury. This was achieved by inhibiting the NOD-like receptor protein 3 (NLRP3) inflammatory vesicles and caspase-1 activation and producing IL-1β and IL-18. The findings suggested that ISL attenuated early brain injury following experimental brain haemorrhage by facilitating the Nrf2 antioxidant channel and repressing ROS- and NF-κB-mediated activation of NLRP3 inflammatory vesicle [44].

Subarachnoid hemorrhage (SAH) is a severe and deadly stroke. SAH is less prevalent than ischemic stroke and intracerebral haemorrhage, but its morbidity is higher among the young population, and it involves higher mortality rates [45]. These findings demonstrate that ISL exerts its neuroprotective effects by inhibiting NLRP3 inflammasome activation through suppression of NF-κB p65 nuclear translocation, thereby attenuating neuroinflammatory responses following SAH, suggesting its potential as a therapeutic agent for SAH management [46].

Traumatic brain injury (TBI) represents a severe neurological disorder associated with diverse long-term complications, including depression, epilepsy, and dementia. The pathophysiology of TBI involves complex secondary sequelae characterized by glutamate-mediated excitotoxicity, disruption of ionic homeostasis, oxidative stress, and neuroinflammatory responses [47-49]. Mechanistic studies by Zhang *et al.* have demonstrated the critical role of GSK-3β in modulating inflammatory cytokine release, neuronal apoptosis, and blood-brain barrier dysfunction following TBI. Importantly, ISL has been shown to attenuate neuroinflammation through inhibition of the PI3K/AKT/GSK-3β/NF-κB signaling pathway, thereby suppressing pro-inflammatory cytokine production in TBI models [50]. Furthermore, experimental evidence has indicated ISL administration to significantly reduce the levels of proinflammatory mediators (IL-1, IL-6, and TNF-α) and pro-apoptotic factor Bax, while upregulating anti-apoptotic proteins Bcl-2 and Bcl-xL in TBI-induced rats [51].

### 3.2 Acute Lung Injury

Sepsis, a life-threatening condition frequently triggered by infection, burn injuries, or traumatic events, exhibits rapid progression to septic shock and multi-organ dysfunction, presenting significant clinical challenges in its management. The pathophysiology of sepsis is characterized by an uncontrolled systemic inflammatory response to infection, resulting in substantial global morbidity and mortality rates [52, 53]. Despite recent advancements in the development of potential therapeutic agents for sepsis and its associated complications, it continues to represent a leading cause of mortality worldwide [54]. It particularly causes acute lung injury (ALI), acute liver failure (ALF), and acute kidney injury (AKI). Despite advancements in clinical treatment, ALI continues to affect approximately 50% of sepsis patients, contributing to 30% of sepsis-related deaths in recent years [55-57]. These clinical challenges underscore the critical need for elucidating the precise molecular mechanisms underlying sepsis-induced tissue injury and developing novel pharmacological strategies for its treatment.

Lipopolysaccharides (LPS), a predominant glycolipid component of the outer membrane in gram-negative bacteria, represent a well-characterized endotoxin that triggers pro-inflammatory cytokine expression and plays a pivotal role in sepsis pathogenesis [58]. Chen *et al.* demonstrated that ISL intervention not only significantly downregulated TNF-α and IL-6 gene expression in LPS-induced mouse peritoneal macrophages (MPMs), but also concurrently mitigated systemic inflammatory responses, thereby attenuating LPS-induced pulmonary and hepatic injury in murine models. These protective effects were mechanistically associated with NF-κB pathway inhibition [59]. In a complementary study, Liu *et al.* established that ISL effectively suppressed NLRP3 inflammasome activation in LPS-stimulated RAW264.7 cells. Utilizing an ALI model induced by intranasal LPS administration, their research study further revealed that ISL ameliorated LPS-induced ALI through inhibition of NLRP3 inflammasome assembly, caspase-1 activation, and IL-1β production in pulmonary tissues [60].

### 3.3 Acute Liver Injury

Acute liver failure (ALF) represents a critical hepatic disorder characterized by extensive inflammatory processes. Despite its clinical significance, the development of effective targeted therapies for ALF remains limited. Wang *et al.* demonstrated that ISL confers hepatoprotection against LPS/D-GalN-induced ALF through multiple mechanisms, including enhancement of cellular oxidative stress resistance, attenuation of inflammatory responses and apoptosis, and inhibition of NLRP3 inflammasome activation, mediated *via* the PGC-1α/Nrf2 pathway [61]. Complementary research has revealed ISL to significantly upregulate SIRT1 activity and expression, enhance nuclear translocation of Nrf2, and suppress NF-κB p65 nuclear accumulation in both *in vivo* and *in vitro* models. These findings collectively suggest that ISL may serve as a potential therapeutic agent for mitigating doxorubicin (DOX)-induced hepatotoxicity through SIRT1 modulation [62].

### 3.4 Acute Kidney Injury

Acute kidney injury (AKI) is a serious condition associated with a significant death rate [63]. The mechanisms underlying AKI in sepsis remain poorly understood, and effective preventive treatment during the early stages is still lacking. This condition is characterised by a rapid decrease in renal function. Recent studies have reported that AKI affects up to 5% of hospitalised patients and 30% of critically ill patients. Globally, AKI is associated with an estimated 2 million deaths per year, establishing it as a pivotal concern globally. Tang *et al.* found that ISL could protect against LPS-induced AKI by preventing the induction of NF-κB p65, a key protein involved in inflammation [64]. Other studies have demonstrated that ISL can effectively reduce the induction of inflammatory cytokines *in vitro* and *in vivo*. ISL has also been shown to treat cisplatin-induced AKI by suppressing the expression of FPR2 [65]. Moreover, ISL increased the expression of GPX4 and xCT, the markers of ferroptosis, to attenuate LPS-induced AKI [66].

## 4 ISL AND CHRONIC DISEASES

### 4.1 Metabolic Disorder

The metabolic disorder syndrome is a significant global public health concern, closely linked to obesity and type 2 diabetes mellitus. Since chronic inflammation serves as a major hazard factor for both conditions [67], the use of anti-inflammatory agents holds promise for preventing and treating metabolic diseases. Lee *et al.* found that ISL supplementation significantly reduced insulin resistance (IR) levels and the expression of plasma inflammatory markers. This suggests that ISL may be beneficial for managing obesity-related metabolic diseases, like IR and inflammation [68]. Honda *et al.* reported that ISL diminished inflammation in mouse macrophages by inhibiting the activation of NLRP3, a protein involved in inflammatory responses. Specifically, ISL inhibited caspase-1 activation and IL-1β secretion [69]. Furthermore, in mice fed a high-fat diet, ISL improved obesity and type 2 diabetes by reducing the accumulation of inflammatory cells and inflammation in adipose tissue. They subsequently identified the effects of transplantation of ISL-modified microbiota on phylum *Sclerotinia*, *Lactobacillus*, and *Lactococcus* in recipient mice, suggesting that the anti-inflammatory and anti-metabolic ramifications of ISL can be overcome by modifying the bacterial composition of the gut [70].

### 4.2 Diabetic Complications

Diabetes has evolved into a global health challenge in recent years. Persistently high levels of blood glucose are a key feature in individuals with diabetes and can contribute to a range of adverse medical outcomes [71]. The most prevalent and serious complication of diabetes is diabetic nephropathy (DN), which has been verified as a major cause of persistent nephropathy and end-stage kidney failure [72]. The pathological manifestations of DN are characterized by a spectrum of renal alterations, including tubular epithelial cell dysfunction, progressive glomerulosclerosis, cellular apoptosis, inflammatory cell infiltration, and tubulointerstitial fibrosis [73]. Recent evidence strongly suggests that inflammation and oxidative stress have an important effect on the advancement of DN [74, 75]. Huang *et al.* found that ISL inhibited the activation of the MAPK pathway in high-glucose environment-stimulated NRK-52E cells and demonstrated the effectiveness of SIRT1-mediated ISL, leading to an improvement in the downstream MAPK-related inflammation and Nrf2-related oxidative signaling pathways [76]. Sun *et al.* found that ISL down-regulated the JAK2/STAT3 signalling pathway and inhibited the expression of downstream inflammatory proteins to safeguard the kidneys of rats in a DN model [77].

Diabetes mellitus is associated with both microvascular and macrovascular complications, representing distinct pathological entities. Microangiopathy demonstrates a direct correlation with the severity and duration of hyperglycemia, whereas macrovascular complications constitute the predominant cause of diabetes-related mortality [78]. Accordingly, reducing macrovascular complications is a crucial aspect of diabetes management, which can improve the quality of life, lower healthcare costs, and mitigate diabetes-related mortality. With this in mind, the development of new, effective treatments to achieve these goals is vital. Alzahrani *et al.* described the protective effects of ISL on aortic injury in STZ-induced diabetes, where ISL maintained the integrity of the aorta and inhibited apoptosis of vascular endothelial cells. These effects were attributed to ISL’s significant inhibition of inflammatory signaling and ROS generation [79].

Diabetic retinopathy (DR) represents a significant neurovascular complication of diabetes mellitus, characterized by progressive degeneration of retinal neurons, glial cells, and vascular elements, ultimately posing substantial threats to visual function. Retinopathy develops to varying degrees in diabetic patients over a span of 20 years and is characterized by retinal ischemia caused by hyperglycemia-induced progressive capillary occlusion [80]. Nevertheless, the exact mechanism of hyperglycaemia-induced retinal rupture is still ambiguous. Additionally, current therapeutic approaches, for example, anti-vascular endothelial growth factor (VEGF) or laser photocoagulation, have drawbacks, such as high cost and potential side effects [81]. These limitations highlight the necessity for alternative or complementary treatment approaches.

Rubler *et al.* first described diabetic cardiomyopathy (DCM) as progressive cardiac dysfunction in diabetes, independent of ischemia or hypertension [82]. DCM pathogenesis involves inflammation, oxidative stress, autophagy defects, mitochondrial dysfunction, endoplasmic reticulum stress, fibrotic remodeling, and apoptotic cell death [83, 84]. There is mounting evidence that inflammatory responses and oxidative stress are significantly activated by hyperglycaemia in the early stages of DCM, and that they may negatively impact the structure and function of the heart. Accordingly, targeting inflammatory and oxidative pathways is crucial for developing new therapeutic approaches for diabetic cardiomyopathy. Gu *et al.* found that ISL effectively restrained cellular hypertrophy, fibrosis, and apoptosis induced by hyperglycaemia. The observed effects were mediated through attenuation of inflammatory responses and reduction of oxidative stress in H9c2 cardiomyocytes. Complementary *in vivo* investigations demonstrated that ISL exhibited significant anti-inflammatory and antioxidant properties, leading to reduced myocardial hypertrophy, fibrosis, and apoptosis, thereby maintaining cardiac function. Moreover, ISL is a promising natural product for the therapy of DCM, with the MAPK and Nrf2 signaling pathways representing effective targets for the prevention and treatment of DCM [85].

### 4.3 Tuberculosis

*Mycobacterium tuberculosis*, the causative agent of tuberculosis (TB), continues to pose substantial global health challenges, with epidemiological data from 2020 indicating approximately 10 million incident cases and 1.5 million mortality events attributable to this disease [86]. The disease progression and clinical outcomes are largely determined by the equilibrium of host immune responses. Infection with *M. tuberculosis* typically induces a robust localized inflammatory reaction, which plays a crucial role in both disease pathogenesis and the immunopathology associated with TB development [87]. While conventional anti-TB therapeutics primarily target bacterial eradication, their clinical utility is often limited by dose-dependent toxicity, adverse effects, and the emergence of drug resistance due to suboptimal patient adherence. These limitations have prompted the development of alternative therapeutic strategies, particularly host-directed therapy (HDT), which may avert the development of bacterial resistance; it has been advocated as a new approach to combat TB by enhancing host defence mechanisms and modulating excessive inflammation, thus reducing infection or augmenting clinical outcomes [88]. Lots of studies have shown ISL to have an inhibitory effect on inflammatory factors, suggesting its potential as a therapeutic agent for HDT. A study by Sun *et al.* used an *in vitro* model of Mtb H37Ra infection and proved that ISL decreased Mtb-induced inflammatory response through Notch1/NF-κB and MAPK signalling pathways. It can be discovered from these findings that ISL could be a latent adjuvant in tuberculosis treatment by modulating the host immune response [89].

### 4.4 Eye Diseases

Glaucoma serves as one of the major causes of vision damage worldwide [90]. The most dominant treatment procedure over the past 40 years has been trabeculectomy. This therapeutic intervention aims to alleviate elevated intraocular pressure (IOP), a primary pathological manifestation of glaucoma. Nonetheless, after a long follow-up period, the success rate of trabeculectomy has been found to decline from an initial 81.2%–85% to 65%–39.3% [91-94]. Conjunctival fibrosis after trabeculectomy has been found to be a dominant reason for failure, with its pathogenesis including overexpression of the renin-angiotensin system, inflammation, proliferation, and vascular regeneration [95-98]. Ye *et al.* found that ISL alleviated angiotensin II (ANG II)-induced fibrogenesis in human trabecular fibroblasts (HTFs) by restraining the NF-κB/PPARγ inflammatory pathway. It can be summarized from their findings that ISL can be a prospective therapeutic agent for conjunctival fibrosis. Moreover, the NF-κB/PPARγ signalling pathway may be a useful target for preventing and treating conjunctival fibrosis following glaucoma surgery [99]. The nosogenesis of age-related macular degeneration (AMD) is also related to chronic inflammation [100] and can induce acute macular degeneration through oxidised low-density lipoprotein (oxLDL)-induced retinal pigment epithelial cell death. Gnanaguru *et al.* studied the effect of ISL on oxLDL-induced NLRP3 inflammatory vesicle activation in human retinal pigment epithelial cells. Their investigation further revealed that oxLDL induced significant cytotoxicity in both primary human fetal retinal pigment epithelial cells and ARPE-19 cell lines through NLRP3 inflammasome activation. Notably, ISL demonstrated cytoprotective effects by inhibiting NLRP3 inflammasome signaling, suggesting its potential in attenuating AMD progression [101].

### 4.5 Cardiovascular Disease

The pathophysiology of acute myocardial infarction (AMI) involves a complex interplay of multiple mechanisms, including inflammatory cascades, oxidative stress, genomic instability, programmed cell death, calcium dyshomeostasis, and mitochondrial impairment [102]. Pharmacological studies have demonstrated ISL's capacity to attenuate oxidative damage and inflammatory responses [103]. Specifically, in AMI models, ISL was found to activate the Nrf2/HO-1 antioxidant pathway, thereby counteracting ischemia/reperfusion-induced oxidative injury. Furthermore, ISL treatment mitigated myocardial inflammation through NF-κB pathway inhibition, resulting in decreased production of proinflammatory cytokines (IL-1, IL-6, TNF-α) and chemokines (MIP1α, MIP2), ultimately reducing infarct size and improving cardiac function [104].

Pulmonary hypertension (PH) is clinically characterized by increased pulmonary arterial pressure, vascular remodeling, and right ventricular hypertrophy, culminating in progressive heart failure [105, 106]. Although the complete molecular pathogenesis of PH remains to be fully elucidated, substantial evidence implicates inflammatory mechanisms as crucial regulators of PH progression [107]. Experimental investigations have revealed ISL to suppress monocrotaline-induced inflammatory responses, normalize pulmonary arterial smooth muscle cell (PASMC) proliferation in both *in vivo* and *in vitro* models, and inhibit hypoxia-mediated Akt phosphorylation. These findings establish ISL's therapeutic potential in chemical-induced PH through modulation of inflammatory pathways and PASMC proliferation dynamics, providing a mechanistic foundation for its clinical application in PH management [108].

Atherosclerosis is the process of plaque formation [109], and it has been identified as a chronic vascular inflammatory condition [110]. Clinical studies have clearly demonstrated that modulating inflammation can prevent atherosclerosis and reduce its associated adverse effects [111], suggesting that an inflammation-focused approach to treatment may be more effective [112]. Previously, Du *et al.* identified the effects of ISL administration on ApoE-deficient mice in reducing lipid levels and inflammatory factor levels, inhibiting oxidative stress, and effectively suppressing atherosclerotic plaque development [113]. Moreover, ISL mitigated NLRP3-mediated vascular endothelial cell pyroptosis *via* activation of SIRT6, suggesting that SIRT6 could be a prospective therapeutic target for ISL in treating atherosclerosis (AS) [114].

### 4.6 Chronic Liver Disease

Alcoholic liver disease (ALD) represents a progressive hepatic disorder characterized by a spectrum of pathological stages, ranging from initial steatosis to alcoholic hepatitis, cirrhosis, and ultimately, end-stage liver failure [115, 116]. Despite the escalating global prevalence and mortality rates associated with ALD, current therapeutic interventions remain limited in efficacy [117]. Experimental evidence demonstrated ISL to significantly reduce hepatomegaly, improve biochemical parameters, and attenuate histopathological alterations in liver tissue. Mechanistically, ISL enhanced fatty acid oxidation through upregulation of PGC-1α-mediated expression of PPARα, CPT1α, and ACADs, while simultaneously suppressing oxidative stress and pro-inflammatory cytokine production (TNF-α, IL-1β, IL-6) [118]. Liu *et al*. revealed that ISL ameliorated hepatic inflammation and fibrosis through dual inhibition of the SPHKs/S1P/IL-17 pathway and ANXA2/STAT3 axis, offering novel therapeutic targets for liver fibrosis management [119].

Non-alcoholic fatty liver disease (NAFLD), the most prevalent chronic liver disorder globally, affects approximately 25% of the worldwide population, underscoring its significant public health impact [120]. The multifactorial pathogenesis of NAFLD involves complex interactions between inflammatory processes and oxidative stress, which are central to disease progression [121, 122]. Zhang *et al.* identified IQGAP2 as a direct molecular target of ISL, subsequently activating the SIRT1 signaling pathway. This IQGAP2-SIRT1 axis modulates lipid metabolism by upregulating PPARα-mediated fatty acid oxidation while inhibiting SREBP-dependent lipogenesis, thereby attenuating systemic inflammation and preventing hepatic fibrogenesis. These findings highlight ISL's therapeutic potential in diet-induced hepatic steatosis and associated metabolic complications [123].

### 4.7 Neurological-related Diseases

Alzheimer's disease (AD), a chronic progressive neurodegenerative disorder, represents the most prevalent form of dementia and is characterized by severe cognitive deterioration. The pathogenesis of AD is marked by two predominant pathological features: neuroinflammation and oxidative stress [124]. Experimental evidence has demonstrated ISL to exert neuroprotective effects by attenuating Aβ oligomer (AβO)-induced inflammatory responses and oxidative damage in BV2 microglial cells, while simultaneously protecting N2a neuronal cells from AβO-mediated neurotoxicity through modulation of the Nrf2/NF-κB signaling pathway, suggesting its potential as a novel therapeutic agent for AD [125]. A complementary research study by Lee *et al.* has elucidated the anti-inflammatory mechanisms of ISL in LPS-stimulated BV-2 microglial cells, revealing its capacity to suppress the production of pro-inflammatory mediators, including nitric oxide and cytokines, through inhibition of ERK/p38/NF-κB signaling cascades [126].

Parkinson's disease (PD), ranking as the second most common neurodegenerative disorder globally, affects approximately 2% of the population aged over 60 years. With the ongoing demographic shift towards an aging population, the prevalence of PD continues to escalate [127, 128]. This progressive neurological disorder is pathologically characterized by the selective degeneration of dopaminergic neurons in the substantia nigra and the accumulation of Lewy bodies, clinically manifesting primarily as motor dysfunction [129, 130]. While the complete etiopathogenesis of PD remains to be fully elucidated, accumulating evidence suggests that neuroinflammation and oxidative stress play pivotal roles in disease initiation and progression [131-133]. Mechanistic studies by Bai *et al.* demonstrated ISL to exert neuroprotective effects through activation of the Nrf2/NQO-1 signaling pathway, significantly attenuating neuroinflammation and ameliorating neurological deficits in PD models [134]. Furthermore, ISL has been shown to inhibit microglia-mediated neuroinflammation in PD, potentially through modulation of the JNK/NF-κB/AKT signaling cascade. These findings collectively underscore the therapeutic potential of ISL in PD management [135].

## CONCLUSION

**Fig. (1):**
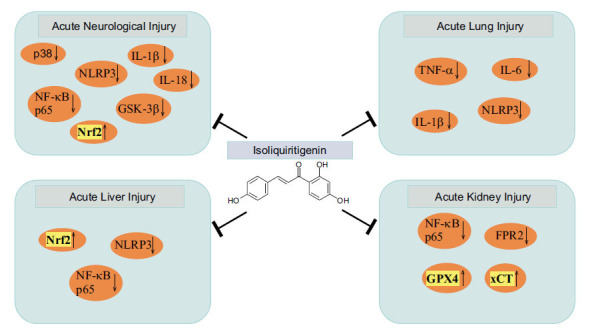
The regulated genes of ISL in acute inflammatory diseases.

**Fig. (2):**
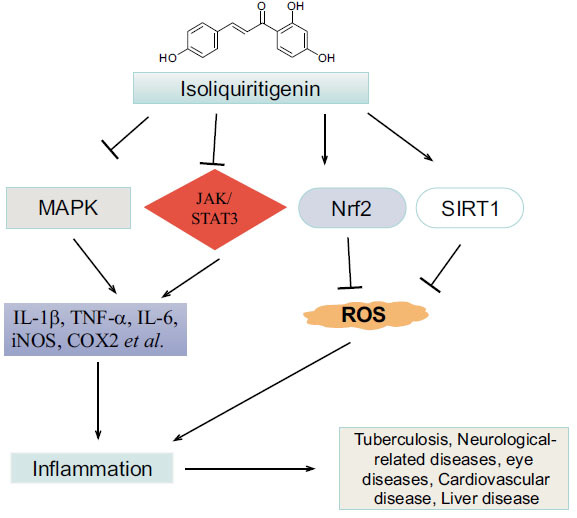
The molecular mechanisms of ISL in chronic inflammatory disease.

This review has highlighted the promising anti-inflammatory potential of isoliquiritigenin, a flavonoid compound with a chalcone structure. Isoliquiritigenin has been found to exhibit excellent safety, showing no lethality up to a dose of 6 mg/kg in *in viv*o mouse assays [136]. Moreover, *in vitro* and *in vivo* studies have demonstrated its effectiveness in modulating various inflammatory pathways. In terms of acute inflammation, it can respond quickly and inhibit the release of inflammatory factors (Fig. **1**). For chronic inflammation, isoliquiritigenin has been reported to exert long-term anti-inflammatory effects by continuously regulating inflammatory signalling pathways (Fig. **2**). Whether systemic inflammation or inflammation localized to a single organ, isoliquiritigenin acts with precise intervention. During systemic inflammation, it modulates the immune system as a whole and reduces the overall level of inflammation. In the case of inflammation of a single organ, such as the liver and lungs, isoliquiritigenin acts specifically on that organ and reduces inflammatory damage. However, further in-depth research is necessary to fully explain its pharmacological effects and establish its safety and toxicological profile. Additionally, isoliquiritigenin has been reported to possess inherent limitations, including poor water solubility and low bioavailability. Addressing these challenges necessitates structural optimization of the compound or the implementation of formulation strategies, such as nanotechnology-based delivery systems. Subsequent research should focus on advancing the structural refinement of isoliquiritigenin derivatives, scaling up the production of nanoformulations, and conducting human clinical trials targeting NLRP3 and other relevant pathways to evaluate long-term efficacy and safety. Despite these limitations, isoliquiritigenin is a significant prospective candidate to serve as a therapeutic agent for inflammatory-related diseases and warrants further investigation for clinical translation.

## LIST OF ABBREVIATIONS

AD: Alzheimer's Disease
AKI: Acute Kidney Injury
ALD: Alcoholic Liver Disease
ALF: Acute Liver Failure
ALI: Acute Lung Injury
AMD: Age-Related Macular Degeneration
AMI: Acute Myocardial Infarction
ANG II: Angiotensin II
AP-1: Activator Protein 1
AβO: Aβ Oligomer
COX-2: Cyclooxygenase-2
DAMPs: Danger-Associated Molecular Patterns
DCM: Diabetic Cardiomyopathy
DN: Diabetic Nephropathy
DOX: Doxorubicin
DR: Diabetic Retinopathy
HDT: Host-Directed Therapy
IL-1β: Interleukin-1 Beta
IL-6: Interleukin-6
iNOS: Inducible Nitric Oxide Synthase
IR: Insulin Resistance
IOP: Intraocular Pressure
IRF: Interferon Regulatory Factor
ISL: Isoliquiritigenin
LPS: Lipopolysaccharides
MCT: Monocrotaline
MPMs: Mouse Peritoneal Macrophages
MAPK: Mitogen-Activated Protein Kinase
NAFLD: Non-Alcoholic Fatty Liver Disease
NF-κB: Nuclear Factor-Kappa B
NLRP3: NOD-Like Receptor Protein 3
NO: Nitric Oxide
oxLDL: Oxidised Low-Density Lipoprotein
PAMPs: Pathogen-Associated Molecular Patterns
PASMC: Pulmonary Arterial Smooth Muscle Cell
PD: Parkinson's Disease
PGE2: Prostaglandin E2
PH: Pulmonary Hypertension
PRRs: Pattern-Recognition Receptors
ROS: Reactive Oxygen Species
SAH: Subarachnoid Hemorrhage
TBI: Traumatic Brain Injury
TB: Tuberculosis
TLR: Toll-Like Receptor
TNF-α: Tumor Necrosis Factor-Alpha
VEGF: Vascular Endothelial Growth Factor

## DECLARATION

The authors would like to thank MogoEdit (https://www.mogoedit.com) for English editing during the preparation of this manuscript.
